# Impact of Molecular Weight on Lymphatic Drainage of a Biopolymer-Based Imaging Agent

**DOI:** 10.3390/pharmaceutics4020276

**Published:** 2012-05-23

**Authors:** Taryn R. Bagby, Shuang Cai, Shaofeng Duan, Sharadvi Thati, Daniel J. Aires, Laird Forrest

**Affiliations:** 1 Department of Pharmaceutical Chemistry, University of Kansas, 2095 Constant Ave, Lawrence, KS 66047, USA; Email: tarynbagby@gmail.com (T.R.B.); scai@ku.edu (S.C.); sduan@ku.edu (S.D.); sthati@ku.edu (S.T.); 2 Division of Dermatology, Department of Internal Medicine, University of Kansas Medical Center, 3901 Rainbow Boulevard, Kansas City, KS 66160, USA; Email: daires@kumc.edu

**Keywords:** lymphatic imaging, hyaluronan, fluorescence, nanoparticle, tumor metastasis

## Abstract

New lymphatic imaging technologies are needed to better assess immune function and cancer progression and treatment. Lymphatic uptake depends mainly on particle size (10–100 nm) and charge. The size of carriers for imaging and drug delivery can be optimized to maximize lymphatic uptake, localize chemotherapy to lymphatic metastases, and enable visualization of treatment deposition. Toward this end, female BALB/c mice were injected subcutaneously in the hind footpad or forearm with a series of six different molecular weight hyaluronan (HA) near-infrared dye (HA-IR820) conjugates (*ca.* 5–200 nm). Mice were imaged using whole body fluorescent imaging over two weeks. HA-IR820 fluorescence was clearly visualized in the draining lymphatic capillaries, and in the popliteal and iliac or axillary lymph nodes. The 74-kDa HA-IR820 had the largest lymph node area-under-the-curve. In contrast to prior reports, mice bearing limb tumors exhibited three-fold longer retention of 74-kDa HA-IR820 in the popliteal node compared to mice without tumors. HA conjugate kinetics and disposition can be specifically tailored by altering their molecular weight. The specific lymphatic uptake and increased nodal retention of HA conjugates indicate significant potential for development as a natural biopolymer for intralymphatic drug delivery and imaging.

## 1. Introduction

Many cancers metastasize via the lymphatic system, including melanoma, breast, colon, lung, and ovarian cancers [1,2]. Accurate determination of which nodes are involved in initial lymphatic spread plays a key role in prognosis and treatment. In melanoma for example, the five year survival rates are >90% for localized disease with no lymphatic involvement, 40–60% if 1 to 3 local nodes are involved, and 15–20% when distant lymph nodes and extra nodal sites are involved [3]. The clinical method for determining the first draining lymph node or sentinel lymph node (SLN) has not changed significantly since its first use in the 1960s. Sentinel lymph node biopsies have been widely used for clinical staging of melanoma and breast cancer since the early 1990s [4,5,6,7,8]. 

SLN mapping involves injection of dye proximal to the primary tumor before imaging or clinical examination. Dyes include (isosulfan blue, patent blue, or methylene blue) or ^99m^Tc-sulfur colloid singly or in combination. Both dye and colloid drain to the regional lymphatic vessels and regional nodes that fuel the SLNs, which is observed visually for dyes, or with a gamma camera, for ^99m^Tc colloids. Each method has its own disadvantages. Dyes suffer from rapid dispersion and lack of localization to only the SLN, risk of anaphylaxis, and propensity for long term staining of skin. Technetium-99m disadvantages include low resolution and radioactivity risks [4,9,10]. Because accurate SLN and lymphatic mapping is crucial in clinical tumor staging, there is a need for new imaging agents for lymphatic mapping. 

Several nanoparticles have been evaluated for lymphatic imaging, including liposomes, micelles, quantum dots (QDs), and iron nanoparticles [11]. Particulate uptake into lymphatic capillaries after subcutaneous administration depends on size, charge, and hydrophobicity; macrophage uptake depends predominantly on size [12,13]. Due to the natural structure of the clefts and pores along the lymphatic capillaries and the presence of aqueous channels (100 nm in diameter) within the interstitium [14], particles with sizes of 10 to 100 nm are preferentially taken up by the lymphatics [13,15]. In contrast, particles > 100 nm largely remain at the injection site as a depot, and particles < 10 nm are reabsorbed by the blood capillaries. Though it is generally accepted that the pores in blood capillaries in the skin are *ca*. 5 nm in diameter and non-fenestrated, it has been shown that capillaries of rodent footpads are fenestrated with pores of 6 to 12 nm [16,17,18], thus the use of the 10 nm cutoff rather than 5 nm for lymphatic targeting. It also has been shown that particulates carrying a net negative charge have enhanced lymphatic uptake and retention in lymph nodes [13,19,20,21]. 

Fluorescent probes are used extensively for preclinical imaging of research animals and increasingly for image-guided surgeries in human patients. To enhance signal to noise ratios for *in vivo* imaging, the emission of the fluorophores must be in the near infrared (NIR) range (600–1000 nm) where there is minimal autofluorescence of hemoglobin, elastin, collagen, chlorophyll (from food), and various other biological chromophores [22,23]. The two chromophores commonly used for *in vivo* imaging are QDs and NIR organic fluorophores. QDs have several qualities ideal for *in vivo* imaging, including inherently bright fluorescence, narrow emission spectra, high quantum yields, excellent photostability, and small size (5 to 20 nm) [24,25]. However, the principal concern with the use of QDs is the toxicity associated with the use of the heavy metals Cd, Te, and Se in QDs cores’ construction. The size and charge of the QDs dictate their clearance mechanism and residence time *in vivo*. Because of the considerable toxicity concerns, the size of the QDs is limited to less than 6 nm to ensure renal clearance and minimize tissue exposure [22,26]. Larger and negatively to neutrally charged (e.g., PEG coated) QDs have shown significant liver and spleen retention, from 1 to 6 months [24,25]. Thus, even though QDs can be conjugated to therapeutic drugs for theranostic applications, are the correct size, and are photostable in the NIR range, toxicity and bioretention may limit their utility. 

We developed an organic NIR fluorophore-polymer conjugate for *in vivo* imaging based off hyaluronan, which meets many of the ideal qualities for an *in vivo* imaging agent. Hyaluronan (HA) is a natural polysaccharide distributed widely in the extracellular space and approved in the US for intraarticular injections, and to use as dermal fillers, and to replace the vitreous during occular surgery. Anionic polymer HA is non-immunogenic and hydrophilic, and its size can be varied by changing the molecular weight. In addition to meeting the requirements for lymphatic uptake and dissemination, HA’s primary receptors, CD44 and RHAMM, are overexpressed on most malignant cancer cells [27,28,29], including invasive breast cancer and melanomas [30]. Our previous studies of targeted lymphatic delivery using 35-kDa HA-chemotherapeutic conjugates (e.g., cisplatin and doxorubicin) demonstrated improved efficacy, decreased toxicity, and superior pharmacokinetics compared to their standard intravenous counterparts [31,32,33,34,35,36]. Herein, we describe the kinetics and optimization of the lymphatic uptake of a series of different molecular weight (6.4 kDa to 697 kDa) hyaluronan-NIR dye conjugates after subcutaneous injection in healthy and tumor-bearing mice. 

## 2. Experimental Section

### 2.1. Materials

Sodium hyaluronate from microbial fermentation was purchased from Lifecore Biomedical (Chaska, MN, USA) and was used without further purification. All other reagents were purchased from Sigma Aldrich Chemical Co. (St. Louis, MO, USA) or Thermo Fisher Scientific (Waltham, MA, USA) and were ACS grade or better. D_2_O and CDCl_3_ were purchased from Cambridge Isotope Laboratories (Andover, MA, USA). PEG standards were obtained from Scientific Polymer Products (Ontario, NY, USA). B16F10 cells were purchased from the American Type Culture Collection (ATCC, Manassas, VA, USA) and were maintained in DMEM supplemented with 10% fetal bovine serum and under a 5% CO_2_ atmosphere. All animal procedures were approved by the University of Kansas Animal Care and Use Committee.

### 2.2. Synthesis of 5-Carboxypentyl-amino-IR-820

The 6-aminohexanoic acid (2 eq.) was dissolved in 20 mL of dry DMF with 2 eq. of triethylamine, and the solution was stirred under argon for *ca.* 5 min. After the addition of 500 mg (0.47 mmol) of IR-820, the reaction mixture was refluxed at 85 °C for 3 h in the dark. The solvent was removed under reduced pressure, and the 5-carboxypentyl-amino-IR-820 was purified by silica column chromatography using a gradient of 2:1 to 1:1 ethyl acetate: methanol. Identity of the blue 5-carboxypentyl-amino-IR-820 solid was confirmed by ^1^H NMR (400 MHz, Bruker) in CDCl_3_; ^1^H NMR (CDCl_3_, 400 MHz): δ = 8.13 (d, *J* = 8.4 Hz, 2H), 7.98–7.91 (m, 4H), 7.63 (d, *J* = 13.2 Hz, 2H), 7.56–7.52 (m, 4H), 7.35 (t, *J* = 8.0 Hz, 2H), 5.77 (d, *J* = 15.2 Hz, 2H), 4.06–4.00 (m, 4H), 3.05 (q, *J* = 12.4 Hz, 6.4 Hz, 1H), 2.57-2.51 (m 4H), 2.16 (t, *J* = 6.4 Hz, 2H), 2.07 (t, *J* = 7.2 Hz, 1H), 1.88 (bs, 11H), 1.82–1.68 (m, 14 H), 1.62–1.53 (m, 3H), 1.48–1.35 (m, 4 H), 1.28–1.22 (m, 1H). 

### 2.3. Synthesis of HA-IR820 Conjugates

The 5-carboxypentyl-amino-IR-820 was dissolved in ddH_2_O (<20 mL) with *N*-(3-dimethylaminopropyl)-*N*′-ethylcarbodiimide hydrochloride (EDAC, 1.2 eq, dye basis) and 4-(dimethylamino)pyridine (DMAP, 0.5 eq., dye basis), the pH was adjusted to 4.0 to 4.5, and the solution was stirred for approximately five minutes. Sodium hyaluronate (100 mg) was dissolved in 10 mL of ddH_2_O and added to the solution (see Table 1 for molar equivalents of reagents used). The mixture was stirred at ambient temperature (*ca.* 20 °C) in the dark. The reaction was monitored by thin layer chromatography, using 6:4:1 methanol: ethyl acetate: dichloromethane as the mobile phase; the total reaction time was typically 1 to 5 days and increased with HA molecular weight. The HA-IR820 conjugate was dialyzed once against 95% ethanol and twice against ddH_2_O. The purple HA-IR820 conjugate was lyophilized and stored at −20 °C until further use. The ratio of the ^1^H NMR peaks in D_2_O of the acetamide methyl of HA and the third methylene groups on the 5-carboxypentyl-amino linker moiety were used to determine the dye content per HA polymer. 

**Table 1:** Molar equivalents of each reagent used in the conjugation of 5-carboxypentyl-amino-IR-820 (dye) to each MW of HA to form the HA-IR820 conjugates.

HA MW (kDa)	Dye Equivalents	EDAC Equivalents	DMAP Equivalents
6.4	1.5	1.8	0.75
35	3	3.6	1.5
74	10	12	5
132	25	30	12.5
357	50	60	25
697	100	120	50

### 2.4. Size Exclusion Chromatography

Conjugation of IR-820 dye to HA was verified by equivalent size exclusion chromatography (SEC) column retention times using evaporative light scattering (ELSD) and UV detection. HA of the same molecular weights (MW) used in conjugation reactions and PEG standard samples were used to generate a calibration curve, and the retention times of HA-IR820 were compared to that of HA and PEG to determine MW changes post conjugation reactions. All SEC traces were collected using a Shodex OHpak SB-806M HQ column thermostated at 35 °C, with 0.8 mL/min mobile phase of 5-mM acetate buffer adjusted to pH 5. Detection was with a UV detector (Shimadzu LC2010CHT) (λ = 520 nm) and an evaporative light scattering detector (Shimadzu ELSD-LT II, 70 °C and 3.6 bar purified air).

### 2.5. Fluorescence

The HA-IR820 samples were dissolved in ddH_2_O at a concentration of 0.1 mg/mL of IR-820. The maximum excitation and emission wavelengths were determined using a fluorescent spectrophotometer (Shimadzu RF-5301 PC, Columbia MD; Panorama Fluorescence Software). The total fluorescence emission spectrum (λ_ex_ = 700 nm, λ_em_ = 800–900 nm) was determined for the HA-IR820 samples, which was used for the normalization of the *in vivo* imaging data.

### 2.6. *In Vivo* Imaging

All mice were fed a low chlorophyll diet at least one week prior to imaging to decrease food induced organ and skin autofluorescence (Harlan 2918 irradiated diet). The mouse’s hair in the area of interest for imaging was removed 24 h prior to imaging with clippers followed by depilatory cream. Female BALB/c mice (20–25 g, Charles River) (3 per group) were anesthetized under isoflurane, placed on a heating pad to help regulate body temperature, and were injected subcutaneously (s.c.) with 10 μL of a 1-mg/mL 5-carboxypentyl-amino-IR-820 or HA-IR820 (dye basis) in the center of the right hind footpad or on the inner-side of the right front forearm. The mice were imaged on both the dorsal and right sides (for footpad injections) or ventral and right sides (for front forearm injections), both with and without the injection site being covered, using whole body fluorescent imaging (Cambridge Research and Instrumentation Maestro multi-spectrum imager, Woburn, MA, USA) with an excitation filter of 710–760 nm and long pass emission filter of 800–950 nm. The animals were imaged for 7 days in the footpad studies and 14 days in the forearm studies. Images were acquired using the autoexposure function in the imaging software to prevent pixel saturation. 

For the imaging studies in tumor-bearing mice, tumors were developed by injecting a 50-μL suspension of B16F10 cells (1.1 × 10^8^ cells/mL) s.c. into the right hind thigh of female BALB/c mice (20–25 g, Charles River, *n* = 5) under isoflurane anesthesia (Day 1). The mouse’s hair in the area of interest for imaging was removed 24 h prior to imaging (Day 3) with clippers followed by depilatory cream. On day 4, mice with similar tumor volume, shape, and location were chosen for the imaging study. Three mice were anesthetized under isoflurane anesthesia, placed on a heating pad to regulate body temperature, and were injected subcutaneously with 10 μL of a 1.0-mg/mL solution (IR-820 dye basis) of 74-kDa HA-IR820 in the center of the right hind footpad. The mice were imaged on both the dorsal and right sides, both with and without covering the injection site, at predetermined time intervals using a whole body fluorescence imager. The animals were imaged for a maximum of seven days. Images were acquired using the autoexposure function in the imaging software.

### 2.7. Data Analysis

Image analysis was performed using Maestro software (version 2.10; CRi Maestro Caliper Life Sciences: Hopkinton, MA, USA, 2010). Regions of interest were placed over the popliteal and iliac lymph nodes or axillary lymph node package. The total signal (scaled counts/s) intensity values (arbitrary units, AU) were recorded for each region of interest (ROI), for all positions (dorsal, ventral, and right sides), and were plotted versus time in GraphPad Prism (version 4, GraphPad Software Inc., La Jolla, CA, USA, 2007). The total integrated fluorescence was determined for each HA-IR820 sample (0.1 mg/mL IR-820) emission spectrum (λ_ex_ = 700 nm, λ_em_ = 800–900 nm), which was used to normalize the ROIs for each HA-IR820 sample. The normalized ROI *vs.* time graphs were integrated to calculate the cumulative fluorescence over time. In addition, the area under the curve (AUC) was determined from the normalized ROI *vs.* time graphs.


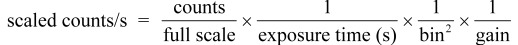


## 3. Results and Discussion

### 3.1. Choice of HA Molecular Weights

Lymphatic uptake is mainly dependent on the size of particles injected into the extracellular space, we chose molecular weights of HA within the optimal size range for lymphatic uptake, 10 to 100 nm. The MWs chosen were 6.4 kDa, 35 kDa, 74 kDa, 132 kDa, 357 kDa, and 697 kDa. Their corresponding sizes (Table 2) were calculated according to the equations derived from Takahashi and his coworkers’ data for radius of gyration (R_g_) and hydrodynamic radius (R_h_) for different MWs of HA in 0.2 M NaCl at 25 °C [37]:


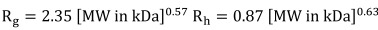


**Table 2:** Radius of gyration (R_g_) and hydrodynamic radius (R_h_) calculated from the fitted equations from Takahashi *et al.* reported data for each MW of HA used in the *in vivo* imaging [37].

HA MW (kDa)	R_g_ (nm)	R_h_ (nm)
6.4	6.77	2.80
35	17.8	8.17
74	27.3	13.1
132	38.1	18.9
357	67.0	35.3
697	98.1	53.8

### 3.2. Polymer Characterization

The synthesis of the reactive IR-820 was adapted from Masotti *et al.* [38] and used to form the HA-IR820 conjugate. The number of IR-820 dyes per HA polymer was determined by ^1^H NMR, and the results are summarized in Table 3. The conjugation efficiency of IR-820 to HA was approximately 30%. The relatively low efficiency can in part be explained by the potential for hydrolysis of the ester bond between the dye and HA upon the dialysis of HA-IR820 against water during the purification process; although, dye hydrolysis was not appreciable during the *in vivo* imaging studies (see below). 

**Figure 1:**
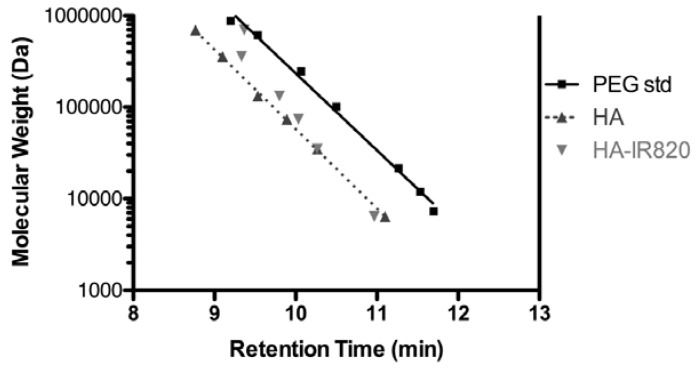
Retention time *vs.* MW for PEG standards, HA samples, and HA-IR820 conjugates from SEC, using a Shodex OHpak SB-806M HQ column, 5-mM acetate buffer (acetic acid/ammonium acetate) at pH 5 (0.8 mL/min flow rate) coupled with UV detection (λ = 520 nm) and evaporative light scattering detection (ELSD, 70 °C).

Conjugation of IR-820 to HA was verified by equivalent elution times using SEC coupled with ELSD and UV detection (data not shown). Linear responses between MW and elution time were observed for both PEG standards and HA samples (*R*^2^ = 0.993 and 0.999, respectively) (Figure 1). Linear polymers of PEG and HA with the same given MW can exhibit dramatically different solution characteristics. HA has a larger excluded volume than that of PEG, which results in the shorter SEC retention time of HA. This is likely due to different conformations of the polymers in solution. HA resides in a stiffened random coil with extensive intermolecular hydrogen bonding; the size is dependent on the solution’s water content, pH, ionic strength and composition [37,39]. However, PEG forms a more compact coiled-coil conformation that presents moderate swelling due to the unique packing of water molecules within the coiled-coil [40]. 

**Table 3:** The loading degree of IR820 onto the different molecular weights of HA that were used for the *in vivo* imaging experiments.

HA MW (kDa)	IR820 wt %	# Dyes/HA Polymer
**6.4**	2.12%	0.16
**35**	4.69%	1.93
**74**	3.21%	2.75
**132**	4.95%	7.72
**357**	4.44%	18.59
**697**	3.97%	32.29

The MWs of the conjugates were similar or slightly lower to that of the original HA used, with the exception of the highest MW of 697-kDa, which exhibited a significantly lower MW than that of the original HA (Figure 1). The smaller MW of the 697-kDa may be due to intramolecular crosslinking mediated by the EDAC [41,42]; a greater concentration of EDAC and longer reaction time was required for this polymer. The resulting crosslinked HA may be more compact and thus have a lower apparent MW by SEC. With the 35-kDa to 357-kDa conjugates, there is some decrease in the apparent MW, as seen in the longer SEC retention times, compared to the original HA, but the change is minimal compared to that of the 697-kDa conjugate.

### 3.3. Spectral Properties

**Figure 2:**
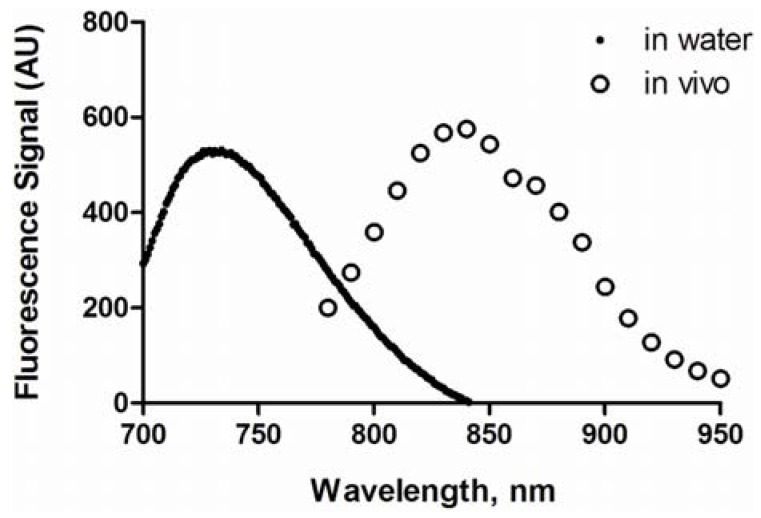
Fluorescence spectra of HA-IR820 conjugates in water and in a mouse.

The maximum excitation and emission wavelengths of HA-IR820 conjugates were determined to be 675 nm and 725 to 735 nm in H_2_O, respectively, with a moderate Stokes shift of 50 to 60 nm. A blue shift in the UV absorbance was observed after each IR-820 reaction (data not shown). The conjugates were diluted to 10% of the concentrations used in imaging experiments to avoid self-quenching, and the maximum absorption wavelengths and total emission spectra were determined over the imaging spectrum of 780 to 950 nm. *In vivo*, the maximum fluorescence emission was at 835 nm (Figure 2). This apparent red shift was due to the improved “optical window” of tissues at higher wavelengths.

For the imaging studies, mice were injected with equivalent amounts of dye and varying concentrations of HA-IR820, since the loading degree of IR-820 varied with the polymer’s MW. If the total concentration was held constant rather than the dye concentration, then the observed differences in the *in vivo* fluorescence signals would include differing concentrations of the fluorophore along with differences in the lymphatic uptake of the various MW HA-IR820 conjugates. Although the dye concentration was held constant, slight variations in the fluorescence intensities were observed *in vitro*. Thus, the *in vivo* fluorescence signal intensities obtained from the ROIs placed over the lymph nodes were normalized using a ratio obtained from the integration of each HA-IR820 conjugate’s resultant fluorescence emission spectra (λ_ex_ = 700 nm, λ_em_ = 800–900 nm). The emission wavelengths of 800–900 nm were used due to the limitations of the spectrofluorometer, whereas the *in vivo* imager recorded the emission from 800 to 950 nm. The MW distribution patterns for the lymphatic uptake and statistical significance between the different HA MWs was unchanged after normalization.

### 3.4. *In Vivo* Imaging

#### 3.4.1. Optimization of Lymphatic Imaging

Selective targeting of the SLNs is possible via s.c. injections into different anatomical regions of rodents [43]. Injection into the hind footpad results in drainage to the popliteal lymph node and then the iliac node via the efferent popliteal trunk. Injection into the front forearm results in drainage to the axillary lymph node package. Because melanoma and breast cancer metastasize primarily via the lymphatics [1,44], identification of the SLN is crucial to staging and treatment of the disease. For localized melanomas of the arms, the murine axillary nodal package corresponds to the human SLN, while the rodent’s popliteal and iliac nodes in the groin correspond to the human popliteal or inguinal lymph nodes involved in malignancies of the legs [45]. Furthermore, the rodent’s axillary lymph node package corresponds to the human SLNs for breast cancer [10,46]. Modeling the SLN drainage kinetics to the popliteal or axillary nodes is instrumental to the development of lymphatically targeted carriers for imaging and drug delivery. 

**Figure 3:**
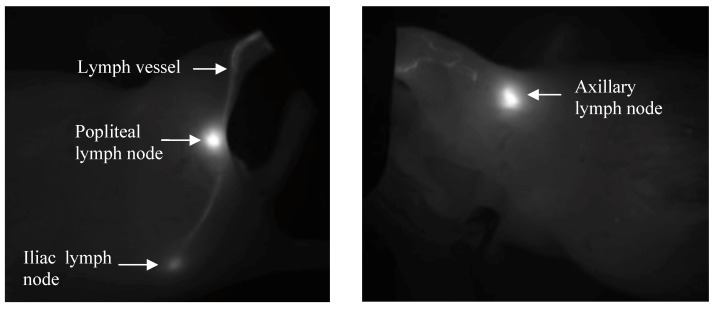
Representative *in vivo* fluorescent images after s.c. injections into the footpad (left panel, upper node is popliteal, lower is iliac), and forearm (right panel, axillary nodes) of HA-IR820 conjugates. Both the lymph nodes and draining lymphatic capillaries can be visualized using *in vivo* imaging with HA-IR820 conjugates. The injection sites were masked in the images in order to visualize the details in the lymphatic drainage.

Dorsal and right lateral images were obtained from mice within the footpad injection group to detect drainage into the popliteal and iliac nodes. Mice in the forearm group were imaged from the right lateral and ventral sides to capture the axillary node drainage. The same trends were observed for both imaging positions, but the right side was analyzed as it produced greater signal intensities and a higher signal to noise ratio compared to the dorsal or ventral views. The ventral position had increased background to signal noise due to the increased fluorescent contributions of the abdominal organs, and the illiac node could not be visualized due to its greater depth from this position. The intense fluorescence signal from the injection site caused pixel saturation in the camera, so the injection site was covered, which enables more sensitive visualization of the draining lymphatic vessels and lymph nodes. For both injection sites, the lymph nodes and draining lymphatic vessels were clearly visualized against the background tissues by fluorescent imaging (Figure 3) with the injection site covered. The auto-expose function resulted in different exposure times for each image; we corrected for these during the image analysis by using the scaled counts per second.

The position of the injection site within the footpad determines where the conjugates will drain. Injections into the center of the hind footpad will drain solely into the popliteal node; however, injections closer to outer edge of the footpad will drain partially into the inguinal node. These observations are in contrast to reports by Ruddell *et al.* stating that the inguinal node is the major draining lymph node from the hind leg [47]. The non-central injections are not representative of the human lymph drainage, since the human foot and leg drain to the popliteal node or the superficial inguinal nodes in the groin area, which corresponds to the popliteal and iliac nodes on a mouse, respectively [43,45]. The human lymph nodes that correspond to the mouse inguinal nodes do not drain the lower limbs in humans. Over the course of 50 injections into the rear hind footpad, the inguinal lymph node was visualized in less than 3% of mice. In addition, bilateral drainage was not observed for any of the subcutaneous injections. Due to the depth limitations of *in vivo* fluorescence imaging (*ca.* 1 cm), only shallow nodes were identified; the authors do not discount further drainage of the HA-IR820 conjugates past the axillary lymph node package or iliac nodes.

#### 3.4.2. Control Experiments

**Figure 4:**
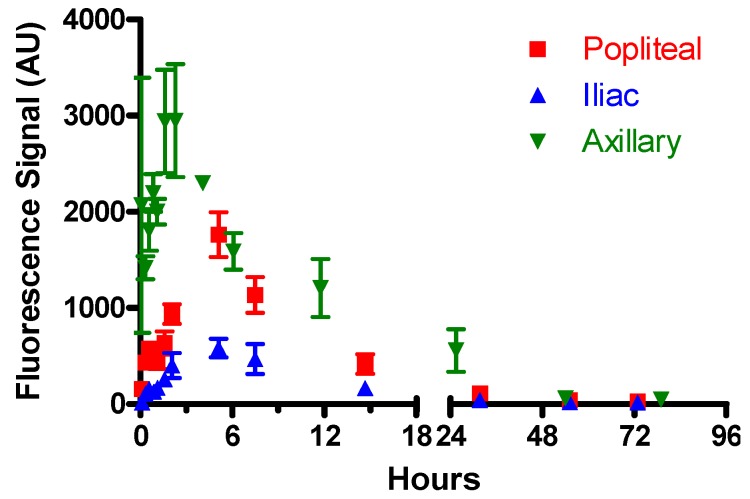
Lymphatic drainage kinetics of the free dye (5-carboxypentyl-amino-IR820) to the popliteal, iliac, and axillary lymph nodes after s.c. injection into the footpad or forearm. Note that the relative fluorescence intensities are proportional to the node depth, thus shallow nodes such as the axillary produce stronger signal intensities resulting in arbitrarily larger fluorescence signals. Relative fluorescence signals cannot be compared between nodes (*i.e.*, axillary, popliteal, and iliac), due to different depths, to correlate relative amounts of the dye present between different nodes.

**Figure 5:**
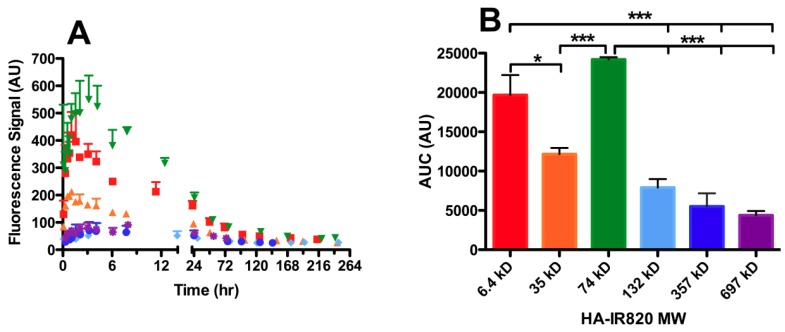
Lymphatic drainage to the axillary lymph node package of different MW HA-IR820 conjugates after s.c. administration. Increased lymphatic uptake of the 74-kDa HA-IR820 conjugate into the axillary lymph node package is evident in the (**A**) time *vs.* fluorescent signal and (**B**) area under the curve (AUC) *vs.* each HA-IR820 MW conjugate bar graph. The MW distribution trends shown are representative of the trends observed for all three lymph nodes (axillary, popliteal, and iliac lymph nodes) visualized after imaging mice on their right sides post HA-IR820 administration (mean ± SEM, * *p* < 0.05, *** *p* < 0.0001).

The trafficking of the unconjugated 5-carboxypentyl-amino-IR820 dye was compared to the conjugated HA-IR820 after injection into both the right hind footpad and the right front forearm. The free dye cleared rapidly from the nodes compared to the HA-IR820 conjugates (Figure 4 and Figure 5A). The free dye was observed in the both the lymph and blood capillaries via *in vivo* imaging (data not shown), whereas the HA-IR820 conjugates were visualized only within the lymphatic capillaries. This indicates that after s.c. injection, the free dye can diffuse into blood capillaries, whereas HA is excluded from hematological uptake within the tissues and is confined to the lymphatic space. More than 75% of the free dye cleared from the lymph nodes within 20 h post injection, and it was completely cleared within 48 h (Figure 4). Similarly, in a study evaluating the lymphatic drainage of free indocyanine green (ICG) and ICG encapsulated liposomes (LP-ICG), both the ICG and LP-ICG cleared within 48 h post injection [48]. Post injection, clinical lymphatic imaging dyes such as isosulfan blue drain rapidly from the injection site. Thirty-four, sixty-nine, and one-hundred percent of the total dye contents are cleared within 30 min, 1 h, and 24 h, respectively [49]. The small degree of retention of the free IR-820 dye after 24 h may be due to its retention within lipid membranes or the formation of conjugates with intracellular proteins.

**Table 4:** The t_max_ and t_50%_ of the lymphatic drainage to the popliteal, iliac, and axillary lymph node packages of different MW HA-IR820 conjugates after s.c. administration.

	t_max_, h			t_50%_, h		
Sample	Popliteal	Iliac	Axillary	Popliteal	Iliac	Axillary
6.4 kDa HA-IR820	1.5	4	1	7.6 ± 3.2	16 ± 7	10 ± 3
35 kDa HA-IR820	1.5	2	1–2	5.7 ± 1.4	4.6 ± 3.6	17 ± 6
74 kDa HA-IR820	1.5–2	1.5–2	3	6.9 ± 2.4	21 ± 1	15 ± 5
132 kDa HA-IR820	1.5	2–4	4	49 ± 15	30 ± 6	62 ± 22
357 kDa HA-IR820	3	3	3–4	38 ± 11	21 ± 9	62 ± 26
697 kDa HA-IR820	3	2–3	3	47 ± 9	62 ± 19	72 ± 8

Mice in the free dye footpad injection group took significantly longer to reach maximal fluorescence (t_max_) compared to the HA-IR820 conjugate groups, but for the forearm injections, the free dye and HA-IR820 groups had a similar t_max_ (Table 4). Because the *in vivo* fluorescence spectra of the free dye (λ_em_ = 823 nm) and HA-IR820 (λ_em_ = 835 and 870 nm) are different, their individual contributions are easily identified after spectral unmixing of the fluorescence signal. The nodal fluorescence signal was due solely to the intact HA-IR820 conjugate and not the free dye. The free dye and the intact HA-IR820 were detected in the livers of mice after the injection of the HA-IR820 conjugates. However, post HA-IR820 injection, free dye was not detected in the lymph nodes, suggesting that the IR-820 conjugates are very stable, and the IR-820 dye is not released until the conjugate progresses through the lymphatics and into the systemic circulation, where it is cleared and degraded by the liver [29]. Again, due to the depth limitations of fluorescence imaging (*ca.* 1 cm) further lymph drainage to the echelon nodes and metabolization within these nodes cannot be detected in live animals. The intact HA-IR820 was detected in the liver 4 to 8 h after administration; however, the fluorescence signal intensity was weak compared to that of the free dye. This suggests the HA-dye conjugates are rapidly degraded once they reach the liver.

#### 3.4.3. Molecular Weight Dependence on Lymphatic Uptake

The dependence of lymphatic uptake on MW was measured using six different HA-IR820 conjugates within the size range reported to have efficient lymph uptake, generally 10 to 100 nm [13,15]. Mice were injected subcutaneously in the center of the hind footpad or in the front forearm with the HA-IR820 conjugates and were imaged at predetermined time points for up to one week in the footpad injections or two weeks in the forearm injections. The drainage of the HA-IR820 conjugates exhibited similar trends for both injection sites and all three lymph nodes; the data presented are for the drainage to the axillary lymph node after injection into the forearm. There was a relationship between MW and lymphatic uptake of HA-IR820 conjugates; the axillary node AUC decreases logarithmically with increasing MW (*R*^2^ = 0.98) when the 74-kDa HA is excluded (Figure 5B and Table 5). Both 6.4-kDa and 74-kDa HA-IR820 have greatly enhanced uptake (large AUC) compared to the other conjugates (Figure 5B and Table 5). Excluding the 6.4-kDa conjugate, there is a Gaussian-type distribution curve over the HA molecular weight range (Figure 5B) for all injection sites, with a maximum at 74 kDa. As the HA MW increased, significant retention in all three lymph nodes was observed compared to the lower molecular weight HA-IR820 conjugates, as indicated by the long t_50%_ values (time required for the fluorescence signal to reduce to 50% maximal) (Table 4). Furthermore, the HA-IR820 conjugates were retained longer in the axillary lymph node package than the popliteal node for all MWs (Table 4). 

**Table 5:** The AUCs of the lymphatic drainage kinetics of different MW HA-IR820 conjugates after s.c. administration into the axillary, popliteal and iliac lymph nodes of BALB/c mice.

HA MW (kDa)	Axillary AUC (AU)	Popliteal AUC (AU)	Iliac AUC (AU)
6.4	19679 ± 2530	9734 ± 1821	3860 ± 466
35	12155 ± 786	3968 ± 1512	1019 ± 425
74	24190 ± 296	11464 ± 1916	4173 ± 1071
132	7897 ± 1101	3589 ± 507	1505 ± 207
357	5514 ± 1669	4887 ± 385	1901 ± 282
697	4381 ± 545	2867 ± 469	1088 ± 375

Although both 6.4-kDa and 74-kDa HA-IR820 had similar lymphatic uptake in all three lymph nodes studied, 74-kDa HA-IR820 was chosen as the optimal MW. Low molecular weight HA (less than 10 to 20 kDa) stimulates angiogenesis [50] and inflammation [51,52], so there may be a different underlying mechanism for uptake of low molecular weight HA, leading to the unexpectedly large AUC of 6.4-kDa HA-IR820. Further investigation into the increased uptake of low molecular weight HA compared to larger HA is underway. The reported optimal liposomal size for lymphatic uptake after s.c. injection is 40 nm [53], which correlates well with our observation that 74-kDa HA-IR820 is optimal for lymphatic uptake (30–50 nm in diameter, based on calculated R_g_ and R_h_ values, see Table 2). In contrast to other polymeric nanoparticle-NIR dye conjugates previously investigated for determining the size dependence on lymphatic uptake utilizing only a few nanoparticle sizes (such as 50 and 100 nm) [54,55,56,57], we investigated the multiple different sized HA conjugates within the relevant size range for lymphatic uptake (10–100 nm). Although these reports also identify the smaller nanoparticles (20–50 nm) as having increase uptake and nodal retention, while the larger nanoparticles remain primarily at the injection site, these polymeric nanoparticle systems do not provide additional insight into the optimal size for lymphatic targeting. It is worth noting, that no signs of toxicity were observed in any of the experimental groups (free dye and all HA-IR820 conjugates) for the entire length of the study, which was up to two weeks in the forearm group). Several animals received multiple injections, and no toxicities or inflammation were observed in animals after as long as 6 months.

A similar effect of MW on lymphatic uptake has been observed with proteins; as the MW of subcutaneously injected proteins increases, the percent recovered dose increases and approaches a plateau at 84 kDa. There was a linear correlation between percent recovered and protein MW up to 30 kDa (83.9% ± 3.3%), which eventually approached 100% with an 84-kDa protein (96.9% ± 6.6%) [58,59]. However, MW was not the only factor governing the lymphatic uptake in those studies, as the charge of the different proteins also varied but was not considered in their analysis. Also, the overall size of the proteins did not differ dramatically, as they were all relatively small (<10–15 nm); however, the size of hydrated volume of HA increases more rapidly with MW. The minimal changes in size of the proteins are due to the compact nature of globular proteins, whereas the large hydrated volume of HA in solution is ascribed to its random coiled structure. Ogston and Stainer described HA as “(behaving) hydrodynamically like a large solvated sphere containing a thousand times more water than organic material” [39]. Reddy *et al.* demonstrated that 71-kDa FITC-labeled dextran has a faster solute velocity within the tissue interstitial space compared to 3-kDa, 40-kDa, and 2-MDa dextran-FITCs or 69-kDa bovine serum albumin [60]. This agrees with our observation that the 74-kDa HA has the greatest lymphatic uptake within the study’s timeframe.

#### 3.4.4. Lymphatic Uptake in the Presence of B16F10 Tumors

Studies of QDs, low MW Gd-DTPA, and indocyanine green liposomes have reported increased lymph flow to the SLN in the presence of B16F10 melanoma tumors [47,48,61]. However, these studies observed the complexes over relatively short timeframes of less than two hours. The lymph flow to the popliteal node in tumor bearing mice was reported to increase up to 23-fold in the 2 min after injection, but after 30 min the flow then decreased to twice the normal flow [61]. Given that our HA conjugates are well-suited as sustained release drug carriers with a t_max_ of 1.5–2 h (74 kDa, Figure 5A and Table 4) and are retained within the lymph nodes for up to five days, tumors are expected to affect the trafficking of HA over much longer timeframes, which would result in greater cumulative egress of HA compared to other materials. Furthermore, the early studies examined flow to the popliteal lymph nodes but excluded successive nodes (e.g., iliac), which would limit the captured kinetic data. 

**Figure 6:**
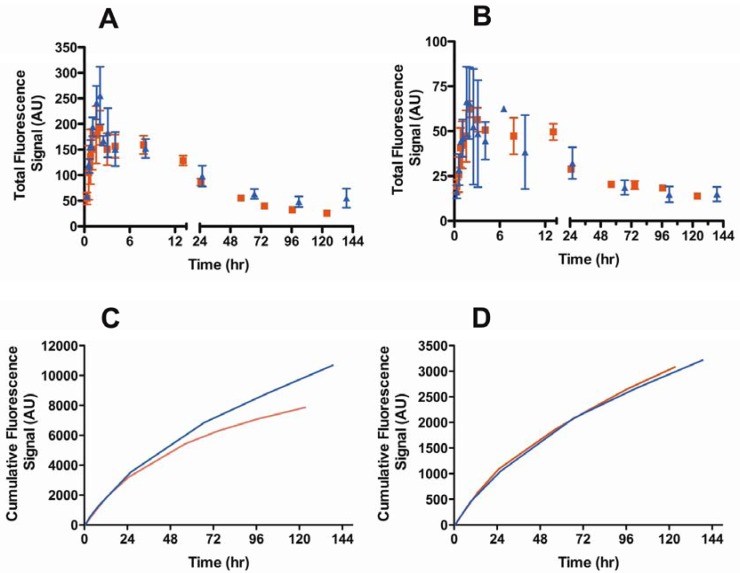
Lymphatic drainage kinetics of the 74-kDa HA-IR820 conjugate into the (**A**) popliteal (*p* = 0.032, student *t*-test) and (**B**) iliac lymph nodes (*p* = 0.58, student *t*-test) in normal (triangles) and B16F10 tumor bearing mice (squares). Cumulative drainage of the 74-kDa HA-IR820 conjugate *vs.* time to the (**C**) popliteal (red line: with tumor; blue line: without tumor) and (**D**) iliac lymph nodes (red line: with tumor; blue line: without tumor) in normal and B16F10 tumor bearing mice.

We examined the effect of a B16F10 melanoma tumor on the lymphatic drainage of both the popliteal and iliac nodes using the optimized 74-kDa HA-IR820 conjugate. The kinetic profiles of the lymphatic drainage to the popliteal node differed significantly between the tumor bearing and normal mice (*p* = 0.0320, student t-test) (Figure 6A); however, the AUC of HA conjugates in the popliteal nodes did not differ significantly between the two groups (Table 6). The drainage from the popliteal node in tumor-bearing mice was almost three times slower than in the normal mice, whereas the drainage to the iliac node remained unchanged (Figure 6B,D, Table 6). Furthermore the t_max_ in the popliteal and iliac nodes was unchanged in tumor-bearing mice compared to normal mice (Table 6). Contrary to the previously published results for liposomes, QDs, and Gd-DTPA, our results indicate similar cumulative uptakes of conjugates into the popliteal node regardless of whether a tumor was present; although, the conjugate drained more slowly from the popliteal node in tumor-bearing animals.

**Table 6:** t_max_, t_50%_, and AUCs for the popliteal and iliac nodes after s.c. administration of 74 kDa HA- IR820 conjugate into the footpad of normal and B16F10 tumor bearing mice.

Lymph Node	Animal	t_max_ (h)	t_50%_ (h)
Popliteal	B16F10 Tumor	2	20 ± 4
	Normal	1.5–2	6.9 ± 2.4
Iliac	B16F10 Tumor	2	23 ± 2
	Normal	1.5–2	21 ± 1

The location of primary tumors in our animals compared to previous reports may partially explain the differences we observed. In the prior studies, the tumor was implanted in the footpad, yet melanomas of the hand are very rare in humans and even less common on the palm [62], which would correspond to the mouse’s footpad. We implanted tumors on the hind thigh, which is more representative of a localized limb melanoma in humans. Furthermore, differently located tumors may induce lymphangiogenesis at different sites, and this too can alter drainage kinetics. The footpad model has been reported to cause aberrant lymph behavior such as induction of long-range lymphangiogenesis within the popliteal lymph node including increased lymphatic sinuses without lymphatic or blood vessel growth within the footpad tumor [61]. Our conjugates may be retained in the tumor tissues longer because they are composed of the naturally occurring polysaccharide HA, rather than artificial materials. HA is a natural ligand for CD44 receptors, which are overexpressed on epithelial, mesenchymal, and lymphoid cells [63]; in addition, CD44 is highly overexpressed on tumor cells and cancer stem cells. While the long retention of these conjugates within sentinel lymph nodes of tumor-bearing animals may not be favorable for imaging when rapid clearance is desired, it may be beneficial in drug delivery applications. Lymphatic uptake of the HA-IR820 conjugate is not impeded by the presence of a large primary tumor, and furthermore the conjugate shows prolonged retention in the sentinel lymph node. Both of these properties may facilitate sustained release of anti-cancer drugs from HA conjugates.

## 4. Conclusions

We optimized the molecular weight of HA for use as a combined carrier for lymphatic imaging and drug delivery. Hyaluronan with a molecular weight of 74 kDa was determined to be optimal for lymphatic imaging due to its maximal lymphatic uptake and enhanced lymph node retention. This corresponds to a size of *ca.* 30 to 50 nm, which is consistent with the reported optimal size for lymphatic uptake of liposomes as well [53]. Previous studies that optimized particulate size for enhanced lymphatic uptake have used either dendrimers between 5 and 20 nm in size, which are so small they are near the size for capillary uptake [64,65], or liposomes and other nanoparticles with non-uniform compositions that vary over an extremely wide size range (100–150 nm spread) [13,53,55]. In addition to variable sizing, the non-uniform particles in prior studies often have very different overall charges or hydrophobicities, and even go so far as to use non-uniform protein compositions [66]. These variables further complicated prior attempts to define the effect of size on lymphatic uptake. We addressed this by using a single polymer, HA, so that resulting differences in uptake kinetics were independent of charge or hydrophobicity. Since HA is a linear polymer, the molecular weight is directly proportional to the number of surface carboxylic acid groups. In contrast, the number of surface groups on dendrimers increases exponentially with molecular weight, and charge can vary with size changes due to increased exposure of the more hydrophobic core.

Hyaluronan is more favorable for clinical imaging than non-biodegradable imaging agents, such as PAMAM dendrimers and QDs. Hyaluronan is used in several clinical products, and we observed no toxicities over several weeks in these studies. Agents such as QDs must be limited to 5 nm or less in size for renal clearance, but even so they are susceptible to prolonged bioretention and heavy metal leakage. In addition, most dendrimers currently under investigation contain multiple amino groups that must be further functionalized with neutral PEG or anionic ligands to reduce their cationic nature and mitigate their toxicity [67]. 

Hyaluronan holds great promise as a natural biopolymer for drug delivery and lymphatic imaging in the clinic. It is non-immunogenic, non-toxic, and it naturally degrades within the lymphatics and liver unlike most other drug delivery platforms. We have shown that the retention of HA within the nodal tissues can be tailored from a period of hours to days; this makes HA conjugates suitable for both sustained drug delivery and long-term imaging of the lymphoid tissues. 
